# Effect of Low-Dose Alcohol Consumption on Inflammation Following Transient Focal Cerebral Ischemia in Rats

**DOI:** 10.1038/s41598-017-12720-w

**Published:** 2017-10-02

**Authors:** Kimberly D. McCarter, Chun Li, Zheng Jiang, Wei Lu, Hillary A. Smith, Guodong Xu, William G. Mayhan, Hong Sun

**Affiliations:** 1 Department of Cellular Biology & Anatomy, Louisiana State University Health Sciences Center-Shreveport, Shreveport, LA USA; 2 Basic Biomedical Sciences, Sanford School of Medicine The University of South Dakota, Vermillion, SD USA

**Keywords:** Cell death and immune response, Diseases of the nervous system

## Abstract

Increasing evidence suggest that low-dose alcohol consumption (LAC) reduces the incidence and improves the functional outcome of ischemic stroke. We determined the influence of LAC on post-ischemic inflammation. Male Sprague-Dawley rats were divided into 3 groups, an ethanol (13.5% alcohol) group, a red wine (Castle Rock Pinot Noir, 13.5% alcohol) group, and a control group. The amount of alcohol given to red wine and ethanol groups was 1.4 g/kg/day. After 8 weeks, the animals were subjected to a 2-hour middle cerebral artery occlusion (MCAO) and sacrificed at 24 hours of reperfusion. Cerebral ischemia/reperfusion (I/R) injury, expression of adhesion molecules and pro- and anti-inflammatory cytokines/chemokines, microglial activation and neutrophil infiltration were evaluated. The total infarct volume and neurological deficits were significantly reduced in red wine- and ethanol-fed rats compared to control rats. Both red wine and ethanol suppressed post-ischemic expression of adhesion molecules and microglial activation. In addition, both red wine and ethanol upregulated expression of tissue inhibitor of metalloproteinases 1 (TIMP-1), downregulated expression of proinflammatory cytokines/chemokines, and significantly alleviated post-ischemic expression of inflammatory mediators. Furthermore, red wine significantly reduced post-ischemic neutrophil infiltration. Our findings suggest that LAC may protect the brain against its I/R injury by suppressing post-ischemic inflammation.

## Introduction

Stroke is the fifth leading cause of death in the United States and the leading cause of permanent disability in adults worldwide. Ischemic stroke accounts for 87% of all diagnosed strokes^1^. Intravenous recombinant tissue plasminogen activator (tPA) and intra-arterial therapy (IAP) are currently used to treat acute ischemic stroke. Both treatments result in a recanalization/reperfusion. Thus, transient focal cerebral ischemia has become one of the most common types of ischemic stroke. Although recanalization/reperfusion is critical for restoring normal function, it can paradoxically result in secondary damage, called cerebral ischemia/reperfusion (I/R) injury^2^. Alcohol is one of the most commonly and regularly used chemical substances. The brain is one of the major target organs of alcohol actions^3^. Epidemiological studies suggest that alcohol consumption has dual effects on both incidence and prognosis of ischemic stroke^4–6^. The mechanisms underlying the dose-dependent beneficial and detrimental effects of alcohol consumption remain uncertain. Wine is one of the three main categories of alcoholic drinks and often used for social drinking and regular light-moderate drinking. Several studies have shown that red wine has a cardioprotective effect^7,8^. However, the influence of red wine on cerebral I/R injury has not been studied. Therefore, our first goal was to determine whether the beneficial effect of low-dose alcohol consumption (LAC) against cerebral I/R injury is dependent on the type of alcohol consumed.

After transient focal cerebral ischemia, acute inflammation subsequently worsens injury in the penumbra area. Post-ischemic inflammation is an important innate mechanism, which is characterized by the accumulation of inflammatory cells and other mediators in the ischemic brain. The recruitment of leukocytes including neutrophils to the ischemic area appears to be a central feature after transient focal cerebral ischemia^9^. When neutrophils are depleted from the circulation the infarct volume is reduced and neurological output is improved^10^. Prior to and during reperfusion, activated cerebrovascular endothelial cells increase their expression of cell surface adhesion molecules that facilitate recruitment of leukocytes to the ischemic area^11^. In addition, the recruitment of leukocytes may be associated with inflammatory activation of microglia. Microglia are the resident immune cells of the central nervous system (CNS) that phagocytose debris under homeostatic conditions as well as injury and disease. When brain injury occurs, microglia rapidly proliferate and transform their morphology. Activated microglia contribute to cerebral I/R injury by producing inflammatory mediators toxic to cells^12^. Cytokines are among the principal mediators of the inflammatory response and are involved in virtually every facet of stroke. Cytokines can be elaborated by leukocytes, macrophages, endothelial cells and resident cells within the CNS. After transient focal cerebral ischemia, altered expression of proinflammatory and anti-inflammatory cytokines worsens cerebral I/R injury^12^. In the heart, the beneficial effect of LAC has been associated with its anti-inflammatory mechanism^8,13^. However, no studies have examined whether LAC has an anti-inflammatory effect in the brain. Therefore, our second goal was to determine the influence of LAC on the inflammatory response following transient focal cerebral ischemia. We further sought to determine whether the type of alcohol consumed is crucial to the anti-inflammatory effect of LAC.

## Results

### Control conditions

Eight weeks of gavage feeding with red wine and ethanol did not significantly alter body weight (Table 1). However, there was a significant reduction in mean arterial blood pressure (MABP) in ethanol group and red wine group when compared to control group. The plasma alcohol concentration was measured at 30 minutes, 1 hour and 2 hours after gavage feeding. The highest concentration appeared at 30 minutes after gavage feeding (Table 1).

**Table 1:** Effect of low-dose red wine or ethanol consumption on body weight, blood pressure and plasma alcohol concentration.

		Control	Red Wine	Ethanol
Body Weight (g)		398.0 ± 9.2	407.3 ± 4.8	399.8 ± 8.9
MABP (mmHg)		118.2 ± 5.8	97.6 ± 7.0*	101.5 ± 5.0*
Blood Alcohol Content (mM)	30′		13.9 ± 0.6	18.8 ± 0.6
	60′		12.1 ± 0.6	15.4 ± 0.6
	120′		6.9 ± 0.7	10.1 ± 0.7

Values are means ± SE for 5–12 rats in each group. *P < 0.05 vs. Control.

### Cerebral I/R Injury

**Figure 1:**
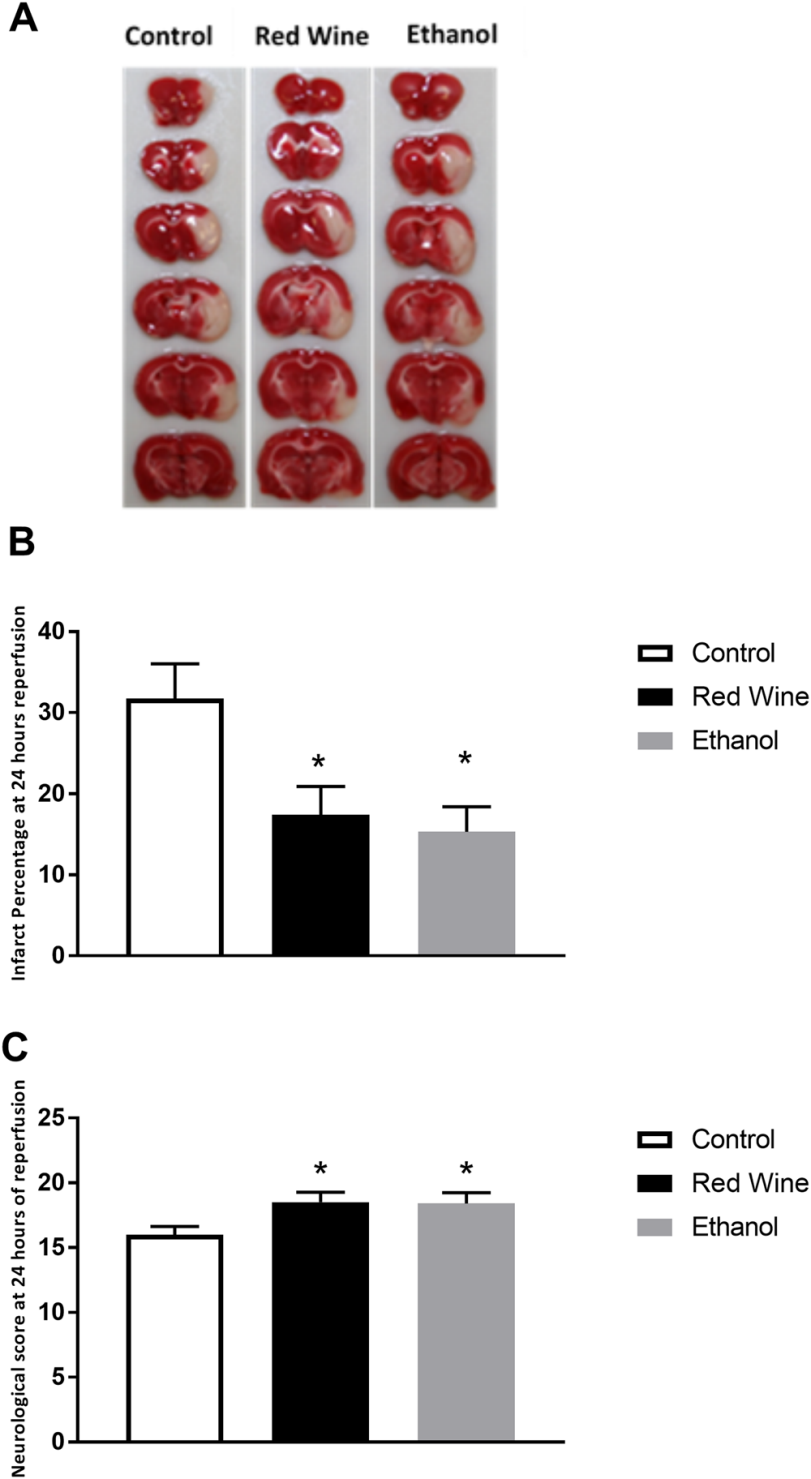
Effect of low-dose red wine or ethanol consumption on brain injury following a 2-hour MCAO/24-hour reperfusion. (**A**) Representative brain sections stained with TTC. (**B**) Total infarct volume. (**C**) Neurological score. Values are means ± SE for 4 rats in each group. *P < 0.05 vs. Control.

There was a significant reduction in total infarct volume in red wine-fed and ethanol-fed rats compared to control rats (Fig. 1). Consistent with the findings regarding the total infarct volume, the neurological deficits were significantly improved in red wine-fed and ethanol-fed rats compared to control rats.

### Protein expression of Adhesion Molecules

**Figure 2:**
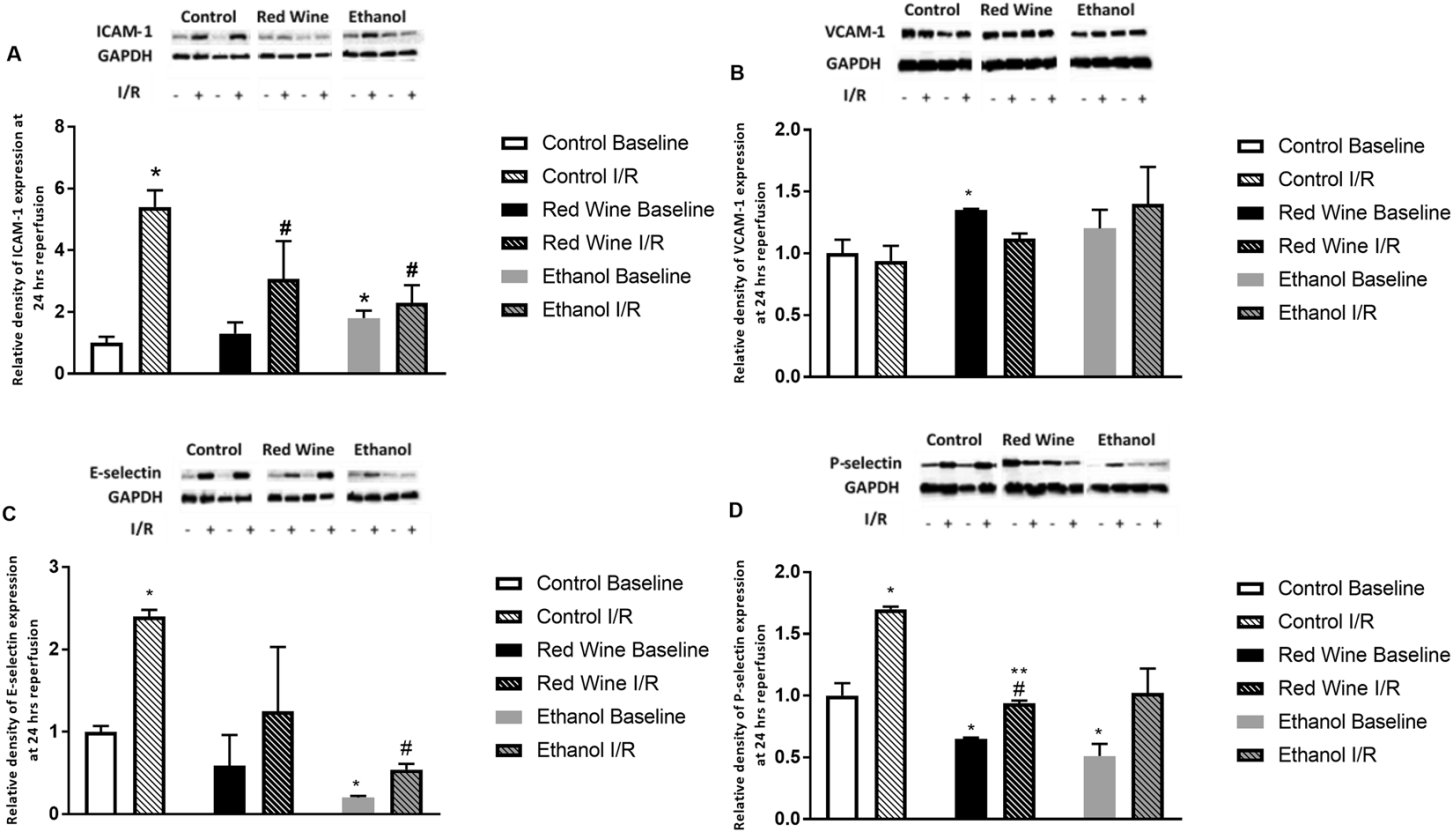
Effect of low-dose red wine or ethanol consumption on expression of ICAM-1 (**A**), VCAM-1 (**B**), E-selectin (**C**) and P-selectin (**D**) following a 2-hour MCAO/24-hour reperfusion. Values are means ± SE for 4 rats in each group. Data shown are representative blots for each group. Ethanol and red wine groups were run on separate gels with two samples from control group as internal control. *P < 0.05 vs Control Baseline. ^#^P < 0.05 vs Control I/R. **P < 0.05 vs Red Wine Baseline.

Both red wine and ethanol significantly attenuated baseline expression of P-selectin (Fig. 2). In addition, ethanol significantly decreased baseline expression of E-selectin. Interestingly, ethanol significantly increased baseline expression of ICAM-1 and red wine significantly upregulated baseline expression of VCAM-1 (Fig. 2). Transient focal cerebral ischemia induced an increase in expression of ICAM-1, E-selectin, and P-selectin but not VCAM-1 in all three groups at 24 hours of reperfusion (Fig. 2). However, the magnitude of the increase in expression of ICAM-1 and P-selectin was significantly less in red wine group compared to control group. On the other hand, the magnitude of increased expression of ICAM-1 and E-selectin was significantly less in ethanol group compared to control group.

### Cytokine Array

**Figure 3:**
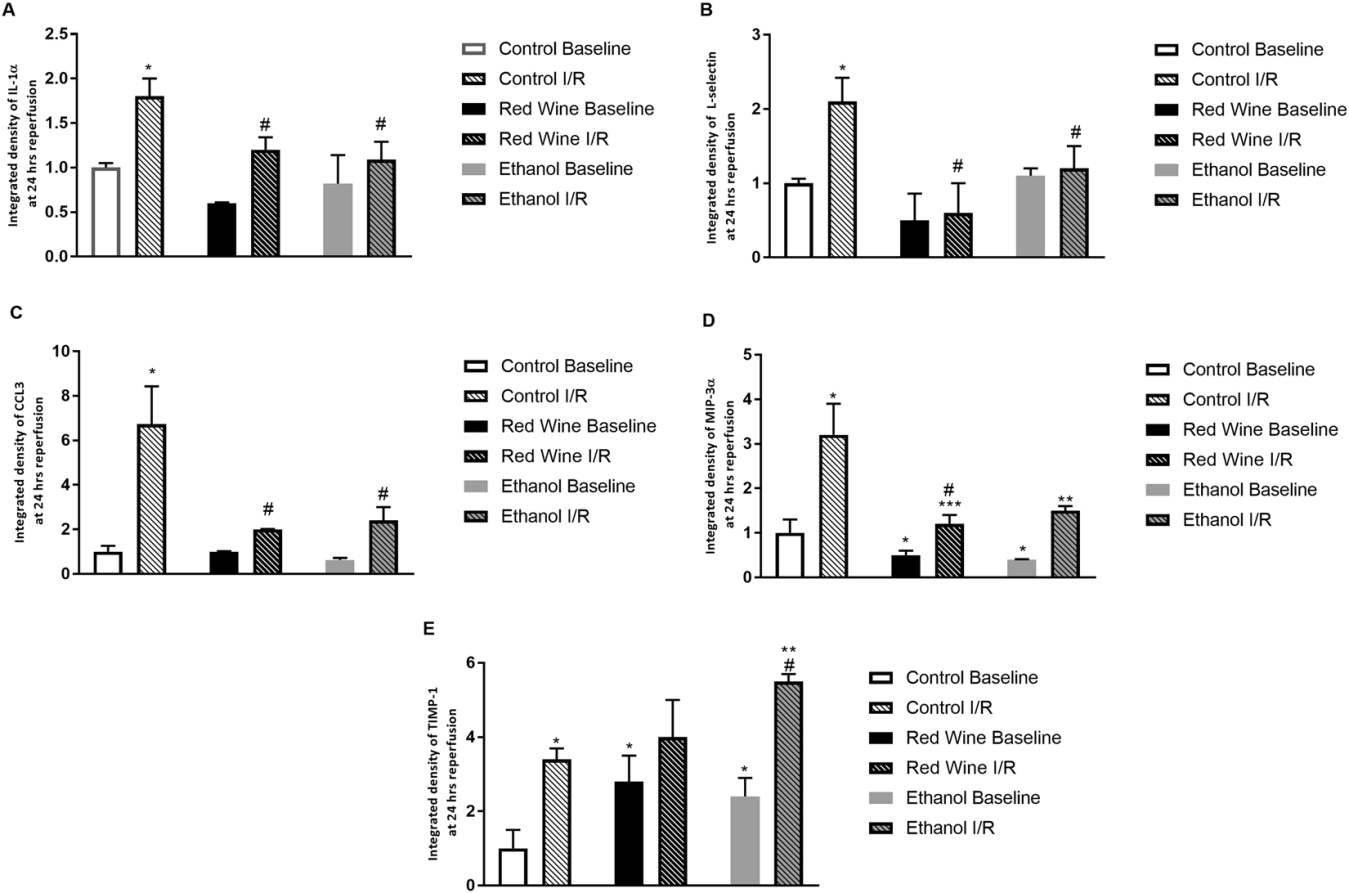
Effect of low-dose red wine or ethanol consumption on expression of IL-1 (**A**), L-selectin (**B**), CCL3 (**C**), MIP-3α (**D**) and TIMP-1 (**E**) following a 2-hour MCAO/24-hour reperfusion. Values are means ± SE for 4 rats in each group. *P < 0.05 vs Control Baseline. ^#^P < 0.05 vs Control I/R. **P < 0.05 vs Ethanol Baseline. ***P < 0.05 vs Red Wine Baseline.

Both red wine and ethanol significantly upregulated baseline expression of TIMP-1 and downregulated baseline expression of CINC-1, IL-6, MIP-3α and CCL17. In addition, red wine also significantly decreased baseline expression of IL-1β, IL-2, CCL5, and VEGF and ethanol also significantly reduced baseline expression of CX3CL1. Two-hour ischemia/24-hour reperfusion produced an increase in expression of IL-1α, L-selectin, CCL3 and MIP-3α in all three groups. However, the magnitude of increase in IL-1α, L-selectin and CCL3 was significantly less in both red wine group and ethanol group (Fig. 3A,B,C). The magnitude of increase in MIP-3α was significantly less in red wine group compared to control group (Fig. 3D). Furthermore, 2-hour ischemia/24-hour reperfusion produced an increase in expression of TIMP-1 in control and ethanol groups, but not in red wine group. The magnitude of increase was significantly greater in ethanol group compared to control group (Fig. 3E).

### Neutrophil Infiltration

**Figure 4:**
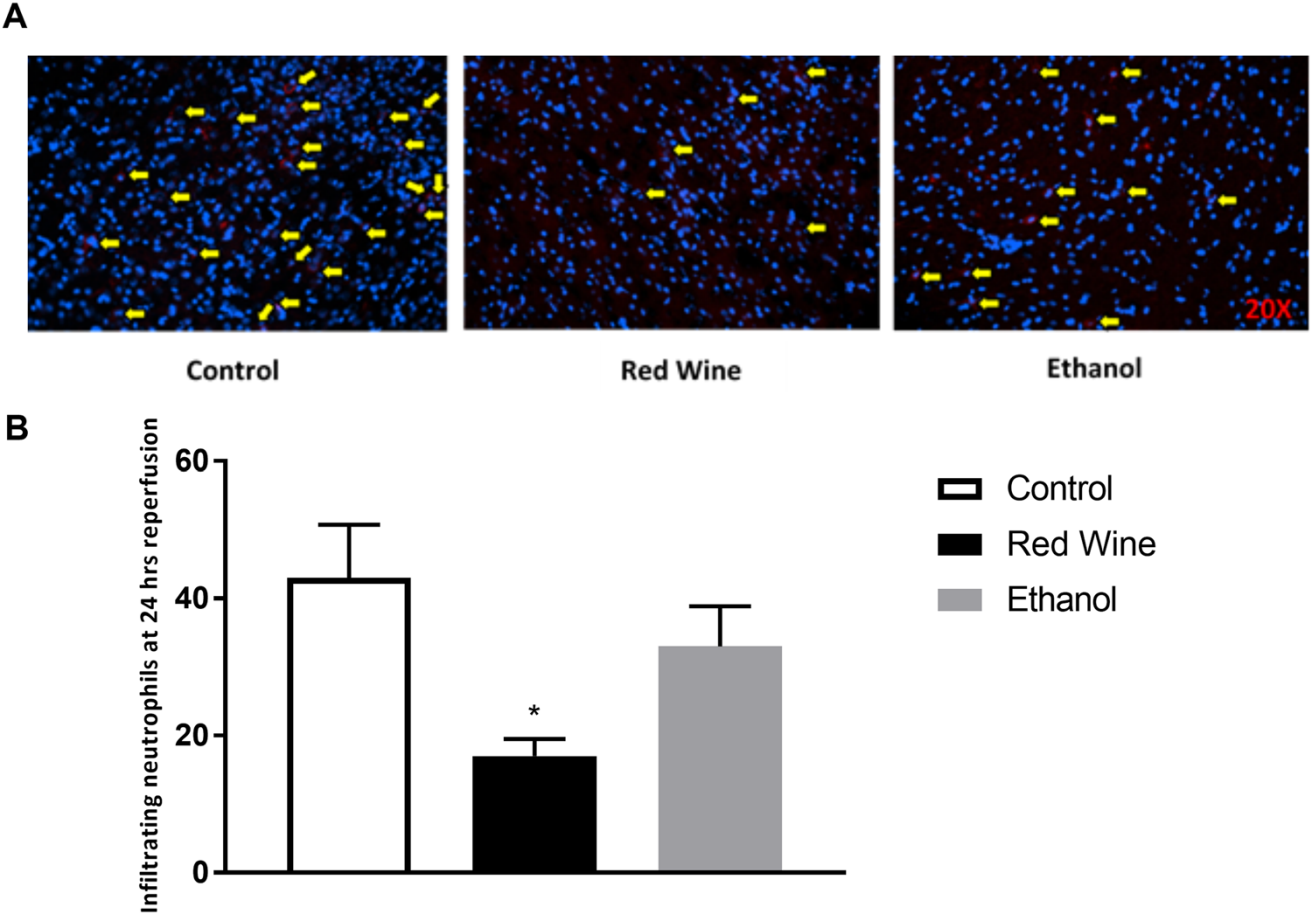
Effect of low-dose red wine or ethanol consumption on neutrophil infiltration following a 2-hour MCAO/24-hour reperfusion. (**A**) Representative MPO staining. (**B**) Values are mean ± SE for 4 rats in each group. *P < 0.05 vs. control.

To examine neutrophil infiltration, positive signals from immunofluorescence staining with MPO were analyzed and counted. There is no neutrophil infiltration detected in the contralateral hemisphere of ischemic brain. Neutrophil infiltration in the ipsilateral hemisphere of ischemic brain was significantly attenuated in red wine group when compared to control group (Fig. 4). Neutrophil infiltration tended to be less in ethanol group, but this did not reach statistical significance (Fig. 4).

### Microglial Activation

**Figure 5:**
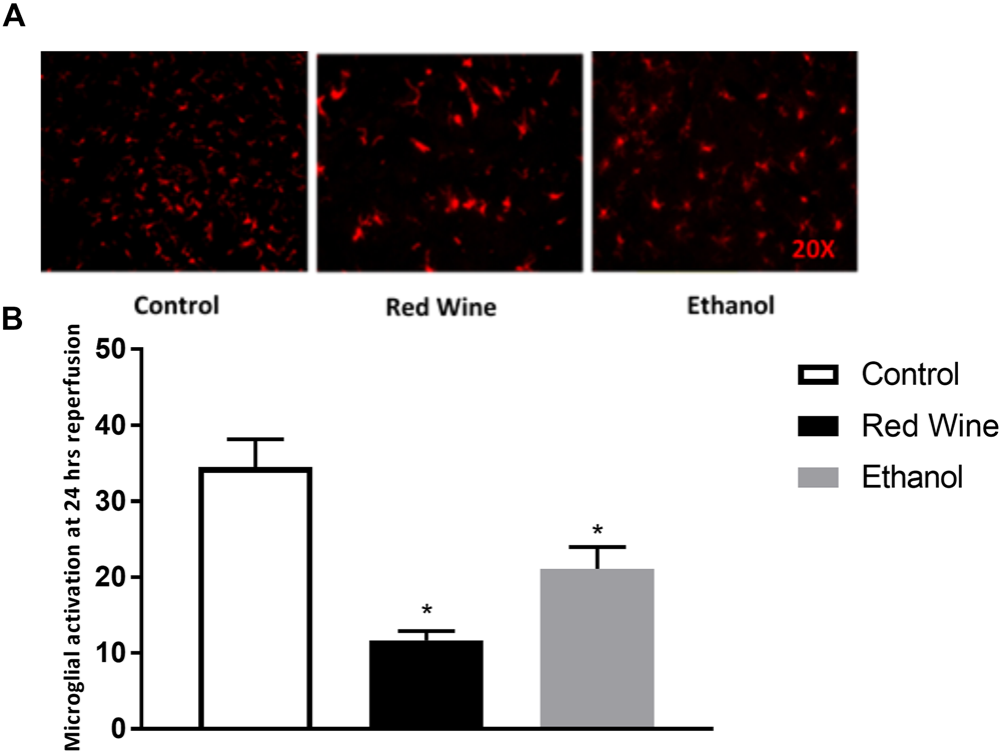
Effect of low-dose red wine or ethanol consumption on microglial activation following a 2-hour MCAO/24-hour reperfusion. (**A**) Representative lba1 staining. (**B**) Values are mean ± SE for 4 rats in each group. *P < 0.05 vs. control.

To assess microglial activation, positive signals from immunofluorescence staining with Iba1 were analyzed and counted. There was no activated microglia detected in the contralateral hemisphere of ischemic brain. Microglial activation in the ipsilateral hemisphere of ischemic brain was significantly reduced in red wine-fed and ethanol-fed rats when compared to control rats (Fig. 5). There was no significant difference in the microglial activation between red wine-fed and ethanol-fed rats.

## Discussion

The present study investigated the influence of low-dose red wine or ethanol consumption on the inflammatory response following transient focal cerebral ischemia. There are several new findings from this study. First, both red wine and ethanol decreased brain damage and improved neurological function following transient focal cerebral ischemia. Second, red wine suppressed ischemia-induced expression of ICAM-1, P-selectin and L-selectin and ethanol delineated ischemia-induced expression of ICAM-1, E-selectin and L-selectin. Third, both red wine and ethanol reduced ischemia-induced elaboration of IL-1α and CCL-3. Fourth, red wine significantly suppressed ischemia-induced infiltration of neutrophils. Fifth, both red wine and ethanol decreased ischemia-induced microglial activation. We speculate that chronic consumption of low-dose alcohol confers a neuroprotective effect against cerebral I/R injury by reducing several factors of the inflammatory response.

Chronic alcohol abuse (more than 3 drinks/day) is considered a risk factor for ischemic stroke, whereas light-moderate alcohol consumption (1 to 3 drinks/day) lowers the risk for ischemic stroke. Furthermore, heavy alcohol consumption worsens the prognosis of ischemic stroke, whereas light-moderate alcohol consumption reduces mortality and infarct volume from ischemic stroke. This dual effect generates a J-shaped pattern in the relationship between chronic alcohol intake and stroke incidence and progress. It is important to note that the beneficial effect of light-moderate alcohol consumption was observed even in those who had other confounding risk factors for stroke such as obesity, hypertension, smoking, and diabetes^14,15^. The average absorption rate of alcohol ranges from 30 minutes on an empty stomach to 90 minutes with food in the stomach^16^. After entering the bloodstream, alcohol travels very quickly to every part of the body. Alcohol is nearly insoluble in fats and oils, although like water, it can readily pass through biological membranes. In addition, there is no plasma protein binding for alcohol. Thus, the concentration of alcohol in a tissue is dependent on the relative water content of the tissue, and reaches equilibrium quickly with the concentration of alcohol in the plasma. The brain is made up of about 75% water and thus is a major target of alcohol actions. Gavage feeding of the rats in the present study increased the plasma alcohol concentration at 30 minutes (Red wine: 13.9 mM; Ethanol: 18.8 mM) after ingestion and then waned until 2 hours (Red wine: 6.9 mM; Ethanol: 10.1 mM) after feeding. The plasma alcohol concentration found in this study corresponded to light alcohol consumption in humans. Therefore, we suggest that our model is appropriate to investigate the chronic effects of low-dose alcohol consumption on the brain.

Numerous studies have investigated the effect of alcohol consumption on cardiovascular diseases. Most studies have focused on the effects of binge drinking, however, some have sought to determine an association between light-moderate alcohol consumption and cardioprotection^17,18^. There have also been a limited amount of studies investigating the protective effects of low-dose alcohol on cerebral ischemia. Wang *et al*. found that acute exposure to ethanol prior to cerebral ischemia produced a protective phenotype in brain microvasculature and neurons^19^. Our previous studies have shown that two-month feeding with a liquid diet containing low-dose alcohol (1%) protected the brain against its I/R injury in rats and mice^20,21^. In the present study, low-dose alcohol was given via an oral gavage, which makes the drinking pattern in our animal model more close to social drinking and regular light-moderate drinking in humans. We found that chronic gavage feeding with low-dose alcohol protected against cerebral I/R injury. Therefore, the results of the present study complement and extend that which we have reported previously. Interestingly, red wine failed to produce a better neuroprotection than ethanol. It seems likely that the neuroprotective effects of LAC are not dependent upon the type of alcohol consumed but rather the amount of alcohol consumed.

As far as we are aware, the present study is the first to investigate the influence of chronic LAC on the post-ischemic inflammatory response in the brain. We found that transient focal cerebral ischemia increased the expression of the adhesion molecules ICAM-1, E-selectin, P-selectin and L-selectin. Interestingly, we did not find an upregulation in VCAM-1 at 24 hours following ischemia. Contrary to our findings, some groups have shown an increase in VCAM-1 expression, whereas one previous study found no change in expression^22,23^. However, Justicia *et al*. found that anti-VCAM-1 treatment did not reduce neutrophil infiltration and was not neuroprotective^22^. Therefore, the role of VCAM-1 in cerebral I/R injury is uncertain. Our results showed that ethanol reduced the magnitude of the increase in expression of ICAM-1, E-selection and L-selectin while red wine reduced the magnitude of the increase in expression of ICAM-1, P-selectin and L-selectin following transient focal cerebral ischemia. The selectins facilitate the diapedesis of leukocytes on the endothelial surface while ICAM-1 mediates the firm adhesion and transendothelial migration of leukocytes. Animals deficient in ICAM-1 or treated with strategies that block ICAM-1 have decreased ischemic damage and less brain neutrophil infiltration^24^. In addition, P- and E-selectin inhibition is associated with improved neurological outcome^25,26^. However, L-selectin’s contribution to cerebral I/R injury is not clear. Of the various types of leukocytes, neutrophils are among the first to infiltrate the ischemic brain^9^. Neutrophils may worsen brain injury in the post-ischemic brain by obstructing capillaries resulting in reduced blood flow during reperfusion as well as by releasing cytotoxic products once they infiltrate into the brain parenchyma. Numerous studies have shown that inhibition of neutrophil infiltration is associated with a decrease in cerebral I/R injury^10,27^. In the present study, neutrophil infiltration was significantly decreased in red wine-fed group and tended to be reduced in ethanol-fed group. Therefore, the neuroprotective effect of LAC against cerebral I/R injury may be related to a reduction in expression of adhesion molecules and subsequent neutrophil infiltration.

Microglia are resident immune cells of the brain and are important modulators of homeostasis and immune response in the brain^28^. These cells are very sensitive to small changes in their environment. At rest, microglia display a ramified state characterized by a small cell body and extensive branches projecting out. Upon stimuli, microglia become activated and undergo several key morphological changes characterized by an amoeboid shape with little to no processes extending out^28^. The penumbra is particularly vulnerable to microglia activation. Activated microglia may contribute to cerebral I/R injury via phagocytosis and elaboration of neuroinflammatory mediators toxic to cells^29^. Studies have shown that decreased microglial activation in the ischemic hemisphere of the brain is associated with marked reduced cerebral I/R injury^30,31^. In the present study, post-ischemic microglia activation was significantly attenuated after chronic low-dose consumption of red wine and ethanol. Therefore, the neuroprotective effect conferred by LAC may be also attributed to a reduction in microglial activation.

Following cerebral I/R, proinflammatory cytokines are elaborated from endothelial cells, leukocytes and resident cells within the brain, including microglia and neurons. These cytokines include IL-1 and TNFα^32,33^. On the other hand, chemokines are chemotactic cytokines and expressed by injured neurons, astrocytes, microglia and endothelial cells following ischemic stroke. Chemokines play a crucial role in the infiltration of leukocytes. Upregulated CINC, CCL-2, CCL-3, MIP-3α, CXCL8 and CX3CL1 are associated with an exacerbated ischemic brain injury^12,34^. In the present study, both red wine and ethanol significantly reduced cerebral I/R-induced expression of IL-1α and CCL-3. In addition, red wine also significantly reduced cerebral I/R-induced expression of MIP-3α. Thus, it is conceivable that the neuroprotective effect of LAC may be related to a decreased post-ischemic expression of cytokines/chemokines. In the present study, we also found that both red wine and ethanol increased baseline expression of TIMP-1 and ethanol further increased cerebral I/R-induced upregulation of TIMP-1. TIMP-1 is an endogenous tissue inhibitor of metalloproteinase. Previous studies have shown that overexpressing TIMP-1 is neuroprotective against ischemic brain injury^35,36^. Thus, it is possible that the neuroprotective effect of LAC may be also related to an alteration in endogenous TIMP.

The French paradox for coronary heart disease (CHD) and wine consumption was described by Renaud *et al*. years ago^37^. This study showed that although there is a high intake of saturated fat in France there was a low mortality from CHD. This was suggested to be attributable in part to regular red wine consumption by residents there. They further demonstrated that the cardioprotection was associated with moderate red wine intake. Since then studies on the benefits of moderate alcohol consumption have been mainly focused on red wine intake with the nonflavonoid components, resveratrol being the most extensively studied. Resveratrol has been shown to be cardioprotective^38^. We previously found that chronic resveratrol treatment protected cerebrovascular function in the type 1 diabetic rats^39^. However epidemiological studies in human patients indicated that other types of alcohol, beer and spirits also are cardioprotective when consumed in moderate amounts. Di Castelnuovo *et al*. and van der Gaag *et al*. demonstrated no significant difference in cardioprotection and the type of alcohol consumed in their studies^40^. We investigated low-dose consumption of both red wine and ethanol to determine if the type of alcohol played a significant role in the neuroprotective effects of LAC on cerebral I/R injury. It is interesting to note that while there was no significant difference between red wine and ethanol in the decrease of infarct size and neurological deficits, red wine and ethanol did show differences in their effect on inflammatory mediators and neutrophil infiltration. In the present study, the red wine group had a faster ethanol clearance rate than the ethanol group. It is conceivable that other active ingredients of red wine, such as polyphenols, may have an anti-inflammatory effect. Therefore, we suggest that both red wine and ethanol are neuroprotective when consumed in low/moderate amounts and this effect is via an influence on post-ischemic inflammation. However, they may differ slightly in the mechanism by which they suppress the inflammatory response.

In the present study, both red wine and ethanol significantly reduced blood pressure. An early study found that a moderate blood pressure reduction prevented brain edema and resulted in a better functional recovery in patients with acute ischemic stroke^41^. However, it remains unclear whether such a reduction in blood pressure is associated with the suppressed inflammatory response. Ethanol is metabolized mainly by alcohol dehydrogenase (ADH), aldehyde dehydrogenase (ALDH), catalase and CYP2E1. Chronic consumption of alcohol may lead to an increase in their activity/expression in the brain^42^. A recent study found that the cardioprotective effect of LAC is related to an upregulated ALDH2^18^. In the future, it will be important to determine the mechanisms by which LAC protects the brain against its post-ischemic inflammation.

In summary, the present study is the first to demonstrate that LAC protects the brain against its I/R injury via suppression of post-ischemic inflammation. In addition, we found that LAC changes baseline immunoprofiles in the brain. This change may be dose-dependent and affect the incidence of ischemic stroke and neurodegenerative diseases. Thus, the present study provides important information for advancing clinical management of transient focal cerebral ischemia in patients who consumes alcohol in mild-moderate amounts. However, further studies are warranted to delineate the specific mechanism by which alcohol influences the inflammatory mediators under basal conditions and following ischemic stroke.

## Methods

### Animal models of Ethanol Preconditioning

All of the procedures and protocols were approved by the Institutional Animal Care and Use Committee at the Louisiana State University Health Science Center-Shreveport and performed in accordance with the National Institutes of Health Guide for the Care and Use Laboratory Animals. Adult male Sprague-Dawley rats (250–300 g) were divided into three groups; control (n = 12), ethanol (n = 12), and red wine (n = 12). Individual group was gavage fed with 13.5% ethanol, red wine (Castle Rock Pinot Noir, 13.5% ethanol) or volume-matched water once a day for eight weeks. The dose of alcohol for the ethanol and wine groups was 1.4 g/kg/day. Plasma alcohol concentration was measured using an Ethanol Assay Kit (ab65343, Abcam). Blood pressure was measured using a CODA rat tail-cuff system (Kent Scientific, Torrington, CT, USA). At the end of 8 weeks of feeding, rats were subjected to transient focal cerebral ischemia.

### Transient Focal Cerebral Ischemia

To avoid a possible effect of acute alcohol, alcohol and red wine were not given on the day before the ischemia. Transient focal cerebral ischemia was induced by unilateral middle cerebral artery occlusion (MCAO) for 2 hours^20^. Prior to the procedure, rats were anesthetized with ketamine/xylazine (100/15 mg/kg ip). A temperature controlled heating pad (Harvard Apparatus, March, Germany) was used to maintain the rectal temperature at 37 °C. A laser-Doppler flow probe was attached to the right side of the dorsal surface of the skull to monitor regional cerebral blood flow (rCBF). The right common and external carotid arteries were exposed and ligated. The MCA was occluded by inserting a monofilament suture (Doccol) into the internal carotid artery to the location where the MCA branches from the internal carotid artery. The onset of the MCAO was indicated by an immediate drop in rCBF. After the occlusion of the right MCA for 2 hours, reperfusion was initiated by withdrawing the suture. Animals were allowed to recover for 24 hours. A 24-point scoring system was used to evaluate sensory/motor deficits at 24 hours of reperfusion^20^. After neurological evaluations, the rats were euthanized with Inactin (150 mg/kg) and the brains were quickly removed. Brain samples from 12 rats (control: n = 4; ethanol: n = 4; wine: n = 4) were placed in ice-cold saline for 5 min and cut into six 2 mm-thick coronal sections. Sections were stained with 2% 2,3,5-triphenyltetrazolium chloride (TTC; Sigma) for 15 min at 37 °C. Sliced images were digitalized, and the infarct lesion was evaluated using ImageJ. The infarct lesion was characterized as an area completely void of staining. Total lesion was expressed as percentage of the ipsilateral hemisphere.

### Western Blot analysis

After neurological evaluations, brain samples from 12 rats (control: n = 4; ethanol: n = 4; wine: n = 4) were isolated to measure protein expression of ICAM, VCAM, E-selectin, and P-selectin. The samples were homogenized in ice-cold lysis buffer containing 150 mmol/l NaCl, 50 mmol/l Tris-HCl, 10 mmol/l EDTA, 0.1% Tween-20, 1% Triton, 0.1% mercaptoethanol, 0.1 mmol/l phenylmethyl sulfonylfluoride, 5 µg/ml leupeptin, and 5 µg/ml aprotinin, pH 7.4. Homogenates were centrifuged at 4 °C for 20 min at 12,000 g and the supernatants were collected. Protein concentration was determined by the Bradford method (Bio-Rad) with BSA as the standard. SDS-PAGE was performed on a 10% gel on which 20 µg of total protein per well was loaded. After SDS-PAGE, the proteins were transferred to a polyvinylidene difluoride membrane. Immunoblotting was performed using mouse anti-ICAM (Santa Cruz Biotechnology), rabbit anti-VCAM (AbCam), mouse anti-E-selectin (Santa Cruz Biotechnology) and mouse anti-P-selectin (Santa Cruz Biotechnology) as primary antibodies and peroxidase conjugated goat anti-mouse and mouse anti-rabbit IgG as the secondary antibody. The bound antibody was detected using enhanced chemiluminescence (ECL) detection (Pierce Chemical), and the bands were analyzed using ChemiDoc MP Imaging System (Bio-Rad). To quantify, protein expression of ICAM, VCAM, E-selectin and P-selectin was normalized to GAPDH and expressed as percentage changes to the control.

### Immunohistochemistry staining

After neurological evaluations, 12 rats (control: n = 4; ethanol: n = 4; wine: n = 4) were anesthetized with Inactin and perfused transcardially with 1X phosphor-buffered saline (PBS), followed by 4% paraformaldehyde in 0.1 mmol/L PBS. The brains were removed, fixed overnight in 4% paraformaldehyde in 0.1 M PBS, dehydrated in a graded series of sugar solutions over the course of 72 hours, and then embedded in O.C.T. compound (Fisher Scientific) and quick frozen for 5 minutes in liquid nitrogen. The frozen brains were then cut into 0.14 µm sections and placed on frost free slides. The sections were washed with 1X PBS, blocked with 10% Bovine Serum Albumin (BSA) for at least 1 hour, and then incubated overnight at 4 °C with 1:100 rabbit anti-MPO (Abcam) for visualization of neutrophils or 1:100 rabbit anti-Iba1 (Wako Chemicals Inc.) for visualization of microglia as primary antibodies. Then the sections were incubated with 1:200 AlexaFluor 555 donkey anti-rabbit (Santa Cruz Technology) for one hour at room temperature. Sections were mounted with DAPI mounting medium with Vector shield and visualized using a fluorescence microscope with a mounted Nikon camera. Cells positive for MPO represented infiltrating neutrophils. For quantitative analysis, positive cells were counted in three separate areas per section surrounding the infarct area in at least three slides per rat in each group. To quantify microglia, cells positive for Iba1 were observed and counted.

### Cytokine Array

Cytokine expression was measured in brain samples from 12 rats prepared for Western Blot analysis as described above. Cytokine expression analysis was carried out using the Proteome Profiler Rat Cytokine Array Kit, Panel A (ARY008, R&D Systems). The array was performed according to the manufacturer’s instructions. Results were analyzed using ImageJ software.

### Statistical Analysis

Data analysis was conducted using GraphPAD prism 7. Data are reported as means ± SE. Differences between groups were evaluated for statistical significance by Student’s t-tests or ANOVA with Tukey’s test where applicable. P ≤ 0.05 was considered to be significant.

## Electronic supplementary material

Supplementary Info 1
