# Author Correction: Effect of Low-Dose Alcohol Consumption on Inflammation Following Transient Focal Cerebral Ischemia in Rats

**DOI:** 10.1038/s41598-019-42589-w

**Published:** 2019-04-17

**Authors:** Kimberly D. McCarter, Chun Li, Zheng Jiang, Wei Lu, Hillary A. Smith, Guodong Xu, William G. Mayhan, Hong Sun

**Affiliations:** 1 Department of Cellular Biology & Anatomy, Louisiana State University Health Sciences Center-Shreveport, Shreveport, LA USA; 2 Basic Biomedical Sciences, Sanford School of Medicine The University of South Dakota, Vermillion, SD USA

Correction to: *Scientific Reports* 10.1038/s41598-017-12720-w, published online 02 October 2017

The original version of this Article contained a typographical error in the spelling of the author Hillary A. Smith, which was incorrectly given as Hillary C. Smith. This has now been corrected in the PDF and HTML versions of the Article, and in the accompanying Supplementary Info 1 file.

The Author Contributions section now reads:

“H.S. and W.G.M. conceived the experiments. K.D.M., C.L., Z.J., W.L., H.A.S. and G.X. conducted the experiments. K.D.M. and C.L. analysed the results. All authors reviewed the manuscript.”

