# Supplementary material for: Effect of Low-Dose Alcohol Consumption on Inflammation Following Transient Focal Cerebral Ischemia in Rats
Source: Sci Rep. 2017 Oct 2;7:12547. doi: 10.1038/s41598-017-12720-w (PMC5624984; doi:10.1038/s41598-017-12720-w)
Supplement: Supplementary file 1 — Supplementary Info 1 [file 41598_2017_12720_MOESM1_ESM.pdf]

Supplementary Information

Effect of Low-Dose Alcohol Consumption on Inflammation Following Transient Focal Cerebral Ischemia in Rats

[1]Kimberly D. McCarter, [1]Chun Li, [1]Zheng Jiang, [1]Wei Lu, [1]Hillary A. Smith, [1]Guodong Xu, [2]William G. Mayhan, and [1]Hong Sun

[1]Department of Cellular Biology & Anatomy
Louisiana State University Health Sciences Center-Shreveport
Shreveport, LA
USA

[2]Basic Biomedical Sciences
Sanford School of Medicine
The University of South Dakota
Vermillion, SD
USA

Corresponding Author: Hong Sun, M.D., Ph.D.
Department of Cellular Biology & Anatomy
LSUHSC-Shreveport
Shreveport, LA
USA
Tel: (318)-675-4566
Fax: (318)-675-5889
E-mail: hsun1@lsuhsc.edu

Supplementary Figure 1

Figure 2A

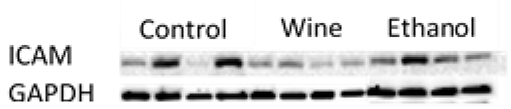

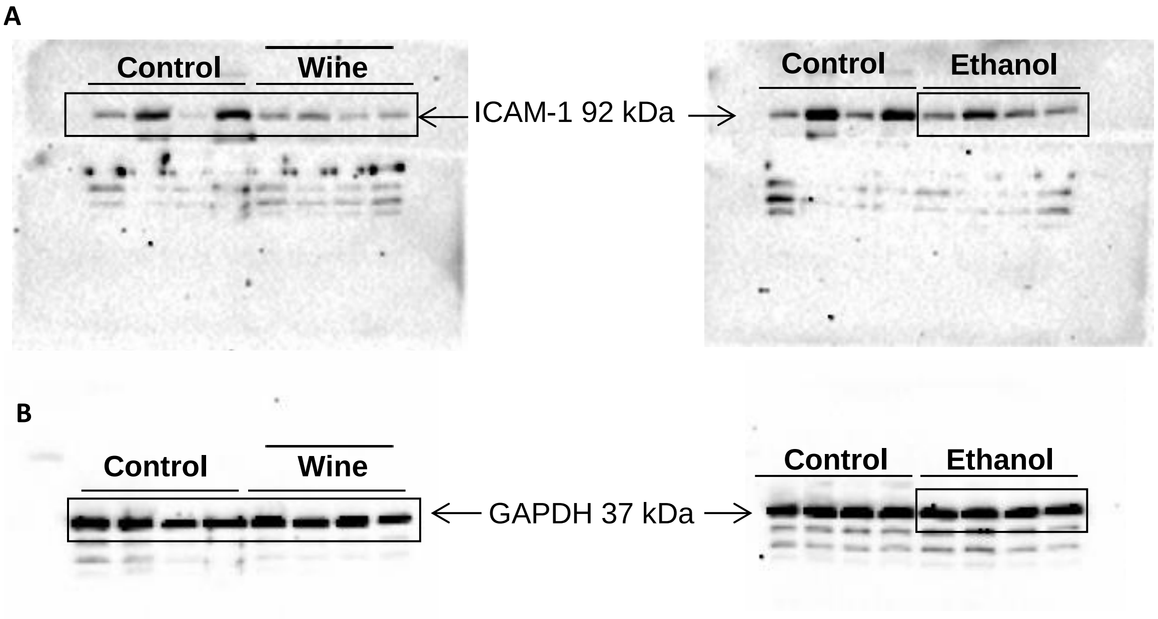

Supplementary Figure 1. Immunoblot membrane images of ICAM expression in control, wine, and ethanol rat groups with and without I/R injury. The cropped images are shown in **Figure 2A**. (A) Immunoblot membrane was cut at ladder 50kDA. The top half probed with anti-ICAM primary antibody while the bottom half was probed with anti-GAPH primary antibody. Both halves were imaged in ChemiDoc MP Imaging System (Bio-Rad) through Chemiluminescence channel. (B) GAPDH failed to be imaged the first time due to investigator error so the bottom half of the immunoblot membrane was stripped and re-probed with the same GAPDH primary antibody and subsequently imaged in ChemiDoC MP Imaging System (Bio-Rad) through Chemiluminescence channel.

Supplementary Figure 2

Figure 2B

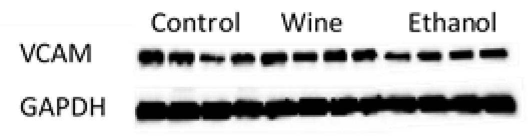

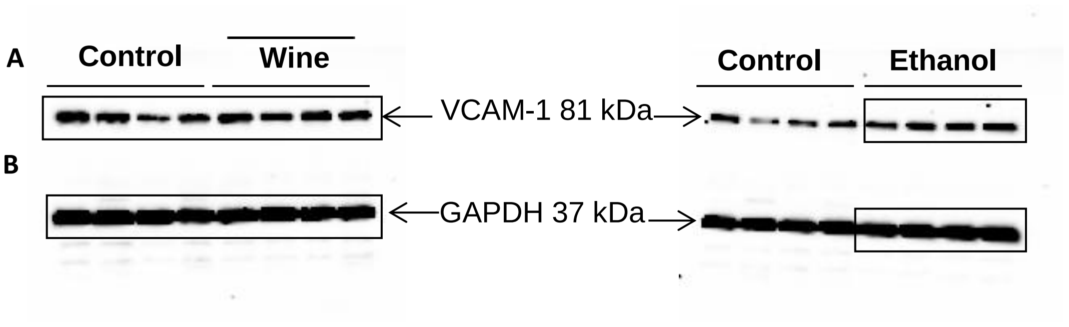

Supplementary Figure 2. Immunoblot membrane images of ICAM expression in control, wine, and ethanol rat groups with and without I/R injury. The cropped images are shown in **Figure 2B**. (A) Immunoblot membrane was cut at ladder 50kDA. The top half probed with anti-VCAM primary antibody while the bottom half was probed with anti-GAPH primary antibody. Both halves were imaged in ChemiDoc MP Imaging System (Bio-Rad) through Chemiluminescence channel. (B) The bottom of the membrane was probed with anti-GAPDH primary antibody and subsequently imaged in ChemiDoC MP Imaging System (Bio-Rad) through Chemiluminescence channel.

Supplementary Figure 3

Figure 3C

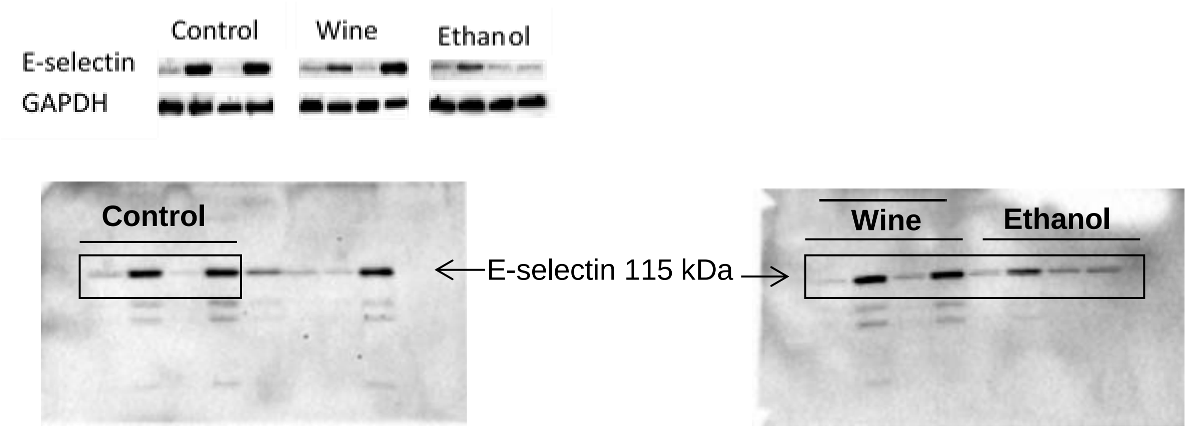

Supplementary Figure 3. Immunoblot membrane images of ICAM expression in control, wine, and ethanol rat groups with and without I/R injury. The cropped images are shown in **Figure 2C**. (A) Immunoblot membrane was probed with anti-E-selectin primary antibody and imaged in ChemiDoc MP Imaging System (Bio-Rad) through Chemiluminescence channel. The band density in these blots were normalized to the GAPDH used for ICAM blots as the samples were used for all blots in this study.

Supplementary Figure 4

Figure 2D

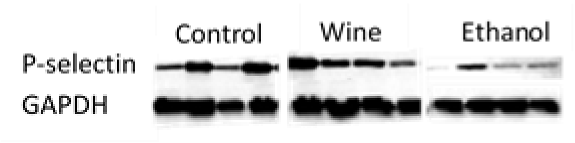

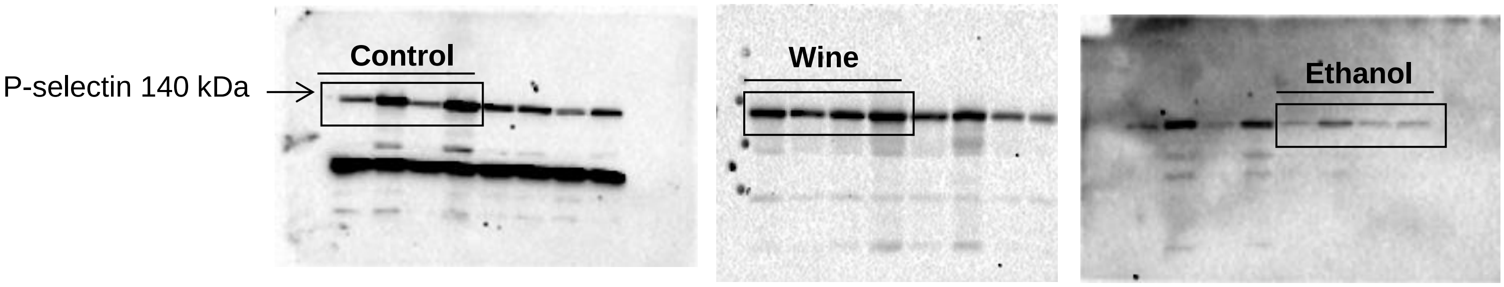

Supplementary Figure 4. Immunoblot membrane images of ICAM expression in control, wine, and ethanol rat groups with and without I/R injury. The cropped images are shown in **Figure 2D**. (A) Immunoblot membrane was probed with anti-P-selectin primary antibody and imaged in ChemiDoc MP Imaging System (Bio-Rad) through Chemiluminescence channel. The band density in these blots were normalized to the GAPDH used for ICAM blots as the samples were used for all blots in this study.
